# Genomic characterization, annotation, and comparative analysis of *Ludisia discolor* reveal its evolutionary and functional traits

**DOI:** 10.1007/s00425-026-04986-0

**Published:** 2026-04-17

**Authors:** Qi Yang, Kunxiu Cai, Junjie Yang, Tianxiang Zhang, Luan Li, Fenfen Wang, Zhendong Chen, Tao Zheng

**Affiliations:** Fujian Institute of Tropical Crops, Zhangzhou, 363001 China

**Keywords:** Chromosome-level genome, Medicinal orchid, Orchid phylogenomics, Orchidaceae, Terpenoid biosynthesis

## Abstract

**Main Conclusion:**

The high-quality *L. discolor* genome clarifies orchid phylogeny and provides immediate targets for breeding and biosynthetic engineering of medicinal metabolites.

**Abstract:**

*Ludisia discolor* is a valued orchid for ornamental veined foliage and documented medicinal uses, yet genomic resources are lacking. We generated the first chromosome-level assembly of 696.37 Mb with scaffold N50 33.14 Mb and 94.55% BUSCO completeness by integrating PacBio HiFi, Hi-C and Illumina reads; 91.86% of sequences were anchored to 22 chromosomes. Annotation yielded 20,552 protein-coding genes and 70.9% repetitive content dominated by LTR-retrotransposons. Comparative analysis revealed 157 species-specific gene families and significant expansion of terpenoid and flavonoid biosynthetic clusters, supporting its pharmacological potential. Phylogenomics placed *Ludisia* sister to *Platanthera* (Orchidoideae) with divergence ~ 39.5 Mya. Positive selection was detected in 123 genes enriched for chromatin remodeling and nuclear transport, reflecting adaptation to shaded, nutrient-limited habitats. This reference genome provides a foundational resource for understanding orchid evolution, molecular breeding, and metabolic engineering of bioactive compounds in *L. discolor*.

**Supplementary Information:**

The online version contains supplementary material available at 10.1007/s00425-026-04986-0.

## Introduction

*Ludisia discolor *(Ker-Gawl.) A. Rich., commonly known as the Bloodleaf Orchid or "*Stone Lotus*" in Chinese, is a perennial herb belonging to the Orchidaceae family. It is native to tropical and subtropical regions of Southeast Asia, including southern China, Thailand, and Vietnam (Dressler 1993). *L. discolor* is renowned for its visually striking foliage characterized by colored veins and reticulated patterns, as well as its chirally asymmetric flowers, making it a popular ornamental plant (Krumov et al. 2022). In traditional medicine, documented in works like *Chinese Materia Medica*, it is used for treating conditions such as tuberculosis, hemoptysis, neurasthenia, and poor appetite (Xu and Wang 2002). Phytochemical studies have identified flavonoids, terpenoids, and phenolic compounds with anti-inflammatory and antioxidant activities (Dipankar et al. 2011; Prommee et al. 2025). Despite its value, genomic studies on *L. discolor* have been lacking, with prior research focusing on phenotypic observations, transcriptomics, and phytochemical analyses. The Orchidaceae is one of the largest angiosperm families, comprising approximately 28,000 species with diverse ecological adaptations. Genomes of several orchids have been sequenced, such as *Phalaenopsis equestris* (Cai et al. 2015), *Dendrobium officinale* (Yan et al. 2015), *Apostasia shenzhenica* (Li et al. 2019), and *Vanilla planifolia* (Gastelbondo et al. 2025), providing insights into flower development, medicinal compound synthesis, and early evolution. However, the genus *Ludisia* remained genomically unexplored. In this study, we assembled the first chromosome-level of the *L. discolor* genome, annotated features, and performed comparative genomics to reveal its phylogenetic position, evolutionary history, and genetic basis for key traits.

## Materials and methods

Details on genome sequencing, survey, assembly and annotations were provided in Supplementary Dataset S1.

## Results

**Fig. 1:**
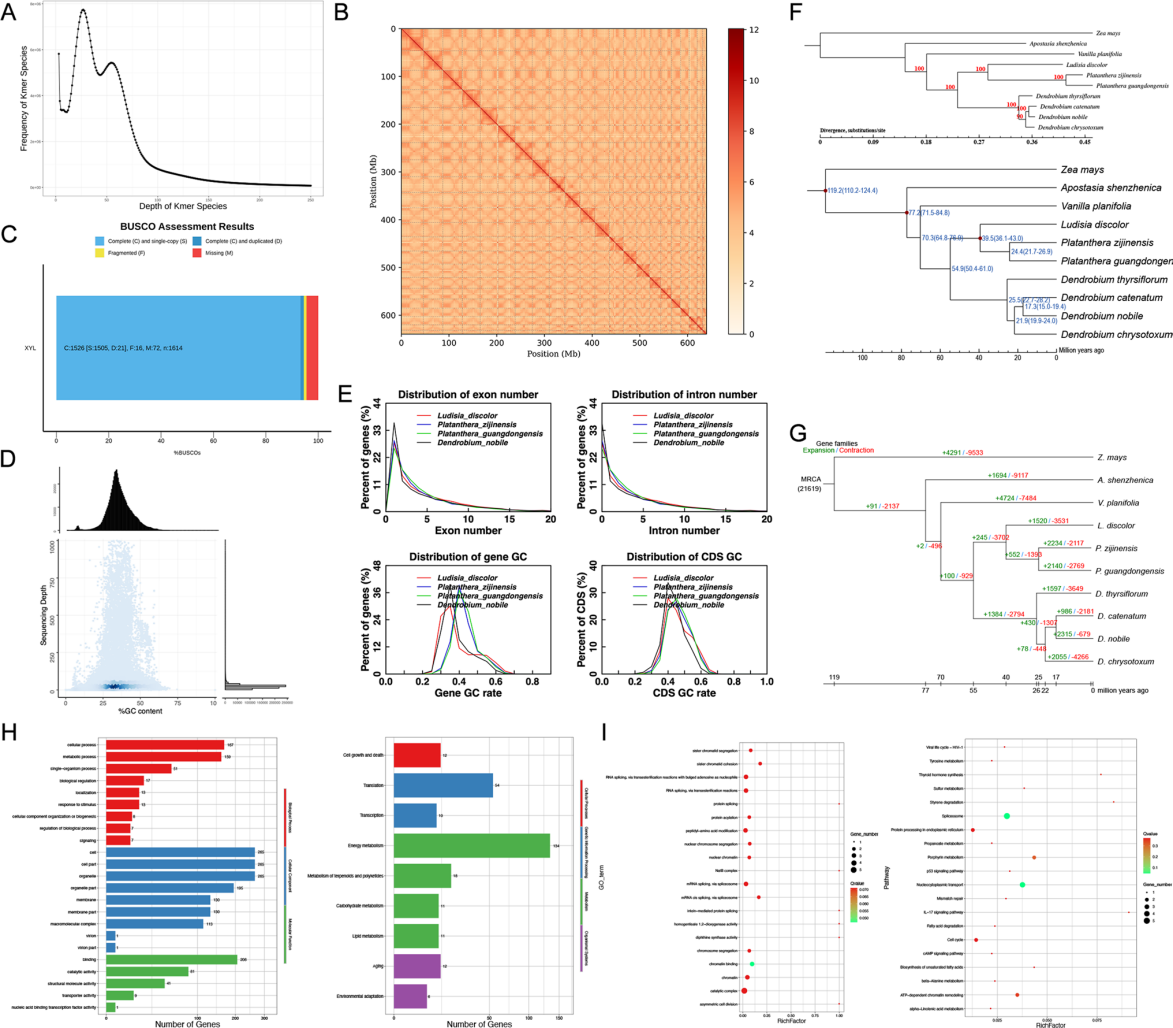
Genome assembly and evolutionary analysis of *Ludisia discolor.* **A** K-mer frequency distribution for genome survey analysis. X-axis represents K-mer depth; Y-axis represents K-mer type count. The main peak (depth = 28) corresponds to the average sequencing depth of the haploid genome; the secondary peak (depth = 14) reflects heterozygous K-mers; the region with depth > 50.4 represents repetitive K-mers. **B** Hi-C interaction heatmap of the chromosome-level genome assembly. X and Y axes represent the 22 chromosomes of *L. discolor* (chr1-chr22), with tick marks indicating genomic positions (Mb). Color intensity reflects Hi-C interaction strength: darker colors indicate stronger cis-interactions (within chromosomes), confirming the accuracy of chromosome scaffolding. **C** BUSCO assessment results of the genome assembly. Bar chart shows the percentage of different BUSCO categories: Complete Single-copy (CS), Complete Duplicated (CD), Fragmented (F), and Missing (M). A total of 1,614 BUSCO genes from the liliopsida_odb10 database were surveyed. **D** GC content and sequencing depth distribution. X-axis represents GC content (%); Y-axis represents average sequencing depth. Point density (color intensity) indicates the number of 1,000 bp non-overlapping windows with specific GC content and depth. The uniform distribution of points confirms no GC bias and consistent sequencing coverage. **E** Protein-coding gene annotation pipeline. The workflow integrates ab initio prediction (Augustus, GlimmerHMM, Genscan), homology-based prediction (using proteins from *P. zijinensis*, *P. guangdongensis*, *D. nobile*), and transcriptome-assisted prediction (RNA-seq), followed by integration using MAKER to generate a non-redundant gene set. **F** Phylogenetic tree and divergence times of 10 monocot species. The tree was constructed using RAxML with 1,000 bootstrap replicates (bootstrap support values > 90% are shown at nodes). Each node shows the divergence time (Mya) and 95% confidence interval (in parentheses). Red nodes indicate calibration points from the TimeTree database. The scale bar represents substitutions per site. **G** Gene family expansion and contraction across 10 monocot species. The phylogenetic tree is consistent with panel **F**. Green numbers indicate expanded gene family counts; red numbers indicate contracted gene f bHLH amily counts. Numbers in parentheses represent gene family counts in the Most Recent Common Ancestor (MRCA). **H** GO and KEGG enrichment analysis of expanded gene families in *L. discolor*. (Left) GO enrichment results, categorized into Cellular Component (CC), Molecular Function (MF), and Biological Process (BP). X-axis represents the number of genes in each GO term; Y-axis represents GO terms. (Right) KEGG enrichment results, grouped by pathway categories. X-axis represents the number of genes in each pathway; Y-axis represents KEGG pathways. All terms/pathways have Q < 0.05. **I** GO and KEGG enrichment analysis of positively selected genes (PSGs) in *L. discolor*. (Left) GO enrichment scatter plot. X-axis represents -log₁₀(Q value); Y-axis represents GO terms (sorted by Q value). Dot size indicates the number of PSGs in the term; color represents enrichment factor (number of PSGs in term/total genes in term). (Right) KEGG enrichment scatter plot. X-axis represents -log₁₀(Q value); Y-axis represents KEGG pathways. Dot size and color follow the same scheme as the GO plot. All terms/pathways have Q < 0.05

We collected fresh *Ludisia discolor* leaves from Fujian, China (24°30'N, 117°30'E) for DNA/RNA extraction via CTAB/TRIzol and utilized Illumina PE150 sequencing (300–500 bp inserts) to generate raw data (46.06 Gb). Details on Genome Sequencing, Survey, Assembly and Annotations were provided in Supplementary Materials (Tables S1-S8). After quality control, 44.86 Gb of clean data were retained with high quality (Q20 ≥ 99.2%, Q30 ≥ 96.8%), with the GC content of both read pairs maintained at 35.5% (Table S1), indicating a reliable genome assembly in the present work. We then conducted K-mer analysis (Liu et al. 2013, K = 17) to estimate genome size, heterozygosity, and repetitive sequence content, key factors for planning de novo assembly. From clean reads, 6.73 billion K-mers were generated, with a main frequency peak at depth 28 (Fig. 1A), reflecting the genome’s average sequencing depth. A smaller peak at depth 14 suggests a heterozygosity rate of 0.63%, typical for perennial herbs and similar to previously reported *Dendrobium officinale* (0.58%, Yan et al. 2015). By comparing K-mer distribution to a Poisson model, repetitive sequences were estimated at 58.02%, common in plant genomes like orchids. This supports using long-read sequencing to handle repetitive regions (Cai et al. 2015). A detailed description on K-mer analyses is provided in Suppl. Dataset S1.

To create a highly continuous genome assembly, we combined PacBio HiFi long-reads with Hi-C data. PacBio Sequel II sequencing produced 418.6 million HiFi read pairs (124.62 Gb total), with an average read length of 148.5 bp and high quality (Q30: 93.8% for R1, 97.2% for R2). Using hifiasm (Cheng et al. 2021), these reads were assembled into 170 contigs, totaling 696.37 Mb, with an N50 of 16.96 Mb and the longest contig at 29.95 Mb (Table S2). Hi-C data were used to build chromosome-level scaffolds. After filtering, 418.6 million clean Hi-C read pairs were aligned to the contigs using BWA-mem2 (Li 2013), with 87.31% alignment (365.5 million reads). Using juicer (Durand et al. 2016), we filtered out 53.1 million invalid read pairs, leaving 290.9 million valid pairs (79.57%) for scaffolding. Using 3D-DNA (Dudchenko et al. 2017) and JuiceBox (Durand et al. 2016), 69 contigs (91.86%, 639.66 Mb) were organized into 22 chromosomes (chr1–chr22). The final scaffold N50 was 33.14 Mb, with chromosome lengths from 7.91 Mb (chr22) to 46.37 Mb (chr1) (Table S2, Fig. 1B).

We then applied three methods to assess the assembly quality, including BUSCO Analysis (Seppey et al. 2019), Read Mapping (Li et al. 2009), and SNP/InDel Detection (McKenna et al. 2010).Details were shown in Suppl. Dataset S1. Overall, the assembly demonstrated high completeness, with BUSCO analysis identifying 94.55% (1,526) of the conserved single-copy orthologs from the liliopsida_odb10 dataset as complete (Fig. 1C). Illumina read mapping showed a high alignment rate (98.26%), with 98.31% of the genome having ≥ 1 × coverage and 93.73% having ≥ 5 × coverage (Fig. 1D). Variant calling using GATK identified 9,718,867 SNPs and 1,077,114 InDels, of which 98.87% and 98.59% were heterozygous, respectively, consistent with the K-mer based heterozygosity estimate and indicating high assembly accuracy.

On the basis of assembled genome, we then annotated the identified repetitive sequences, protein-coding genes and non-coding RNAs. Specifically, to annotate repeat sequences in the *Ludisia discolor* genome, a combination of homology-based (using RepBase with RepeatMasker and RepeatProteinMask) and de novo (using RepeatModeler and LTR-FINDER) methods identified 493.73 Mb of repeats, constituting 70.9% of the 696.37 Mb genome (Table S3). Tandem repeats, detected by TRF (Benson 1999), accounted for 62.16 Mb (8.93%), while de novo TEs contributed 478.51 Mb (68.71%), with long terminal repeat retrotransposons (LTR-RTs) predominant at 358.25 Mb (51.44%), followed by DNA transposons (10.19%), LINEs (3.54%), and SINEs (0.17%) (Table S4), aligning with high LTR-RT prevalence in orchids like *Dendrobium officinale*. Protein-coding gene annotation integrated de novo (Augustus, GlimmerHMM, Genscan), homology-based (Exonerate with protein sequences from related orchids), and transcriptome-assisted (RNA-seq from roots, stems, leaves, and flowers via GMAP, StringTie, and TransDecoder) predictions using MAKER (Holt and Yandell 2011), yielding 20,552 non-redundant genes with an average length of 11,000 bp, CDS length of 1,172 bp, and 5.18 exons per gene (Table S5, Fig. 1E). Functional annotation assigned 93.6% of genes (19,237) to databases, with high coverage in NR (93.43%), TrEMBL (93.23%), KEGG (92.41%), SwissProt (70.92%), and InterPro (73.55%), confirming conserved functions (Zdobnov and Apweiler 2001,Table S6). Non-coding RNAs were annotated using Rfam, INFERNAL, tRNAscan-SE, and BLASTN, identifying 2,754 miRNA genes (0.11% of the genome), 1,300 tRNA genes (0.01%), 2,032 rRNA genes (0.33%), and 63 snRNA genes (Table S7), supporting roles in gene regulation, translation, and mRNA splicing, with no scaRNAs detected, suggesting lineage-specific divergence. These results, consistent with other orchid genomes, validate the reliability of the annotations. A detailed description of these annotations were provided in Suppl. Dataset S1.

We then further explored the evolutionary relationships and molecular adaptations of *Ludisia discolor* through comprehensive genomic analyses. Using OrthoFinder (v2.5.4,Emms and Kelly 2019), we clustered gene families across 10 species, identifying 12,866 gene families in *Ludisia discolor*, including 157 species-specific families potentially associated with unique traits such as colorful leaves and medicinal metabolite synthesis. Compared to *Dendrobium nobile* (14,593 families) and *Zea mays* (13,127 families), *Ludisia discolor* exhibited fewer families and a lower average of 1.474 genes per family (vs. 2.004 in *Zea mays* and 1.848 in *Dendrobium chrysotoxum*), reflecting its smaller 696.37 Mb genome and fewer recent gene duplications (Table S8). We constructed a robust maximum likelihood phylogenetic tree with RAxML (v8.2.12; Stamatakis 2014) using 1,017 single-copy orthologous gene families, achieving > 90% bootstrap support, which grouped *Ludisia discolor* with *Ludisia* A. Rich. (Orchidoideae) as a monophyletic clade, sister to *Dendrobium* (Epidendroideae), with *Apostasia* and *Vanilla* as basal orchids (Fig. 1F), echoed with previous findings (Cai et al. 2015; Yan et al. 2015). Employing PAML (v4.9j; Yang 2007) and r8s (v1.8.1; Sanderson 2003), we estimated the divergence of *Ludisia discolor* from Platanthera at 39.5 Mya (95% CI: 36.1–43.0 Mya), aligning with Oligocene climate-driven orchid diversification (Zachos et al. 2001). Through CAFE (v5.0; De Bie et al. 2006), we detected 1,520 expanded and 3,531 contracted gene families in *Ludisia discolor* relative to the most recent common ancestor (21,619 families), with expansions significantly enriched in GO terms like “cell” (265 genes), “binding” (206 genes), and “metabolic process” (159 genes), and KEGG pathways such as energy metabolism (134 genes) and sesquiterpenoid/triterpenoid biosynthesis (18 genes), vital for medicinal metabolites, while contractions affected pathways like “protein dimerization” and “focal adhesion,” suggesting adaptations to epiphytic lifestyles (Fig. 1G, 1H). Using PAML (Yang 2007), we identified 123 positively selected genes (ω > 1, *P* < 0.05), enriched in GO terms such as “chromatin binding” (Q = 0.023) and “nucleosome assembly” (Q = 0.038), and KEGG pathways including the spliceosome (3 genes, e.g., PRPF8, SNRNP200) and nuclear-cytoplasmic transport (3 genes), indicating targeted optimization of RNA processing and intracellular transport (Cook et al. 2007) to enhance *L. discolor*’s resilience to environmental stresses like temperature fluctuations and nutrient limitations (Fig. 1I).

## Discussion

In summary, this study reports the first chromosome-level genome assembly of *Ludisia discolor*, a medicinal and edible orchid, with a 696.37 Mb genome, scaffold N50 of 33.14 Mb, and 94.55% BUSCO completeness, outperforming many orchid genomes (e.g., *Apostasia* shenzhenica, scaffold N50 = 22.3 Mb). These metrics establish the assembly as a high-quality reference genome, providing foundational resource for future studies in orchid genomics, pigmentation biology, and secondary metabolism, particularly for *Ludisia discolor* breeding, medicinal development, and evolutionary studies within Orchidaceae. Of note, its smaller size compared to *Dendrobium officinale* (1.15 Gb) and *Vanilla* planifolia (1.53 Gb) reflects lower repeat content (70.9% vs. 80%), with 51.44% LTR-RTs supporting transposable element-driven expansion (Kidwell and Lisch 2000). Annotation of 20,552 protein-coding genes and 6,149 non-coding RNAs, with 93.6% functionally annotated, identified 157 species-specific gene families linked to colorful leaves and medicinal traits, and expanded terpenoid/polyketide metabolism genes (e.g., chalcone synthase, CHS) as biosynthetic targets. Phylogenetic analysis placed *Ludisia discolor* closest to *Platanthera* species, diverging at 39.5 Mya during Oligocene cooling, with positive selection on psbA and genes in spliceosome and nuclear-cytoplasmic transport pathways, suggesting adaptive evolution related to shade tolerance and environmental resilience.

Several limitations should be considered when interpreting these results. Although the assembly is highly contiguous, 119 scaffolds (8.14%) remain unanchored, which may limit resolution of fine-scale structural variation and repeat-rich regions. Additional long-read sequencing and/or complementary scaffolding data may further improve anchoring and completeness. Moreover, this work is based on a single reference individual; thus, intraspecific variation, population structure, and recent evolutionary dynamics cannot be evaluated within the current dataset. Future population-level sampling and resequencing will be required to connect genomic diversity with phenotypic variation and to enable robust population genomic inference.

Second, while the genome and annotation provide useful candidate genes and pathway components that may be relevant to pigmentation-related traits, the present work does not include targeted anthocyanin profiling, systematic regulatory network reconstruction, or direct functional validation of pigmentation regulators in *L. discolor*. Accordingly, any references to pigmentation mechanisms are best viewed as resource-oriented observations enabled by genome annotation, rather than definitive mechanistic conclusions. In well-studied model plants, anthocyanin biosynthesis is often regulated by the MYB–bHLH–WD40 (MBW) module (Nesi et al. 2000; Ramsay and Glover 2005; Gonzalez et al. 2008; Xu et al. 2015); however, the systematic identification and experimental interrogation of specific bHLH or WD40 components and their interactions were outside the scope of the current work. Future studies can integrate co-expression/network analyses with protein–protein interaction assays (e.g., yeast two-hybrid and BiFC) to refine potential regulatory relationships (Hu et al. 2002; Walter et al. 2004).

Finally, environmental and developmental influences on pigmentation-associated metabolites were not investigated, reflecting the static, genome-resource design of this study rather than a dynamic physiological framework. Previous reports in other plant systems indicate that high light intensity and low temperature can promote anthocyanin accumulation, and that drought or ultraviolet stress can induce anthocyanin biosynthesis. Because environmental and developmental sampling were not incorporated into the experimental design, physiological regulation cannot be resolved within the current study. Future research will investigate the impacts of these environmental factors on anthocyanin content and regulatory gene expression in *L. discolor*. For instance, shading experiments can clarify the role of light in leaf pigmentation, and quantitative real-time PCR analysis of stress-treated plants can reveal the involvement of anthocyanins in stress responses.

## Supplementary Information

Below is the link to the electronic supplementary material.

Supplementary file1 (DOCX 102 KB)

Supplementary file2 (XLSX 20 KB) Table S1 Quality metrics of Illumina sequencing data for the genome survey of *Ludisia discolor*.Table S2 Assembly statistics of the *Ludisia discolor* genome at the contig and scaffold levels.**Table S3** Summary of repetitive sequences in the *Ludisia discolor* genome.**Table S4** Classification of transposable elements (TEs) in the *Ludisia discolor* genome.**Table S5** Structural features of protein-coding genes in the *Ludisia discolor* genome.**Table S6** Functional annotation statistics of protein-coding genes in the *Ludisia discolor* genome.**Table S7** Annotation statistics of non-coding RNAs (ncRNAs) in the *Ludisia discolor* genome.**Table S8** Gene family clustering results of 10 monocotyledonous plant species.

## Data Availability

All the data generated or analyzed during this study are included in this published article.
