# Supplementary material for: Genomic characterization, annotation, and comparative analysis of Ludisia discolor reveal its evolutionary and functional traits
Source: Planta. 2026 Apr 17;263(5):135. doi: 10.1007/s00425-026-04986-0 (PMC13090214; doi:10.1007/s00425-026-04986-0)
Supplement: Supplementary file 1 — Supplementary file1 (DOCX 102 KB) [file 425_2026_4986_MOESM1_ESM.docx]

Dataset S1. Materials and methods used in this study.

Experimental materials and nucleic acid extraction

Fresh leaves of *Ludisia discolor* were collected from Zhangzhou, Fujian Province, China (24°30′N, 117°30′E). To minimize heterozygosity bias, a single individual was selected for whole-genome sequencing. Genomic DNA was extracted using the CTAB method, and residual RNA was removed with RNase A (Thermo Fisher Scientific, Waltham, MA, USA). DNA quality was assessed using a Nanodrop 2000 (Thermo Fisher Scientific) and an Agilent 2100 Bioanalyzer (Agilent Technologies, Santa Clara, CA, USA), with OD260/280 values required to be between 1.8 and 2.0, and DNA integrity number (DIN) greater than 8.0. Total RNA was extracted from roots, stems, leaves, and flowers using TRIzol reagent (Invitrogen, Carlsbad, CA, USA) to support transcriptome-assisted gene annotation. RNA quality was measured using an Agilent 2100 Bioanalyzer, with RNA integrity number (RIN) required to exceed 7.0.

Illumina short-read library for genome survey

One microgram of genomic DNA was fragmented into 300–500 bp fragments using a Covaris M220 ultrasonicator (Covaris, Woburn, MA, USA). End repair, A-tailing, and adapter ligation were performed using the NEBNext Ultra II DNA Library Prep Kit (New England Biolabs, Ipswich, MA, USA). After PCR amplification, the library was purified using AMPure XP beads (Beckman Coulter, Brea, CA, USA). The final libraries were sequenced on the BGI MGISEQ-2000 platform (BGI, Shenzhen, China) with 150 bp paired-end reads (PE150).

PacBio HiFi long-read library

Ten micrograms of high-molecular-weight genomic DNA were sheared into 10–15 kb fragments using g-TUBEs (Covaris). The SMRTbell Express Template Prep Kit 2.0 (Pacific Biosciences, Menlo Park, CA, USA) was used for fragment repair and adapter ligation. The 10–15 kb fragments were size-selected with AMPure PB beads (Pacific Biosciences). Sequencing was conducted on the PacBio Sequel II platform in CCS mode (≥ 2 passes), generating HiFi reads with an accuracy greater than 99.9%.

Hi-C chromatin interaction library

Nuclei were isolated from fresh leaves and cross-linked with 1% formaldehyde. Chromatin was digested with the restriction enzyme HindIII (New England Biolabs), followed by end repair, biotin labeling, and ligation at 16 °C. The crosslinks were then reversed, and DNA was purified. Biotin-labeled DNA was captured with streptavidin magnetic beads (Invitrogen), fragmented to 300–500 bp, and processed following the standard Illumina library construction protocol. The Hi-C libraries were sequenced on the MGISEQ-2000 platform using paired-end 150 bp reads.

Genome survey and K-mer analysis

Raw Illumina reads were filtered using SOAPnuke (v2.1.0; <https://github.com/BGI-flexlab/SOAPnuke> ;Chen et al. 2018) with parameters set to -lowQual=20 -nRate=0.005 -qualRate=0.5, to remove adapter sequences, low-quality reads (Q < 20), and reads containing >0.5% N bases.

Sequencing quality (base quality, GC content, error rate) was evaluated using FastQC (v0.11.7; <https://www.bioinformatics.babraham.ac.uk/projects/fastqc/>). To detect potential contamination, 10,000 randomly selected clean read pairs were aligned to the NCBI nucleotide (NT) database using BLAST. K-mer analysis was performed using GCE (v1.0.2; <https://github.com/fanagislab/GCE> ;Liu et al. 2013) to estimate genome size, heterozygosity, and repeat content.

A K-mer size of 17 was chosen to balance genome coverage and computational efficiency.

Genome size (G) was calculated based on the Lander–Waterman model.


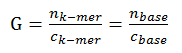


Genome assembly

PacBio read contig assembly

PacBio third-generation SMRT sequencing technology was used to reduce assembly complexity and improve assembly continuity and completeness due to its long reads and lack of GC bias (Chin et al. 2016). The library consisted of dumbbell-shaped circular templates with hairpin adapters at both ends. Raw polymerase reads were obtained by rolling circle sequencing, with low-quality sequences (Q < 20) and adapters removed to yield subreads. Subreads were classified based on pass number: CLR (pass < 2, insert size > 15 kb, error rate 85%–90%) and CCS (HiFi; pass ≥ 2, insert size ≈ 10 kb, accuracy ≥ 99.9%). CLR data were assembled into contigs using NextDenovo (v2.4.0) or MECAT2 (v2.0.0) and polished using gcpp (v2.0.2, PacBio tools) or Pilon (v1.22; Walker et al. 2014). HiFi reads were assembled into contigs using hifiasm (v0.15.1-r334; Cheng et al. 2021) with default parameters. This method exploits the high accuracy of HiFi reads to generate continuous contigs without additional error correction.

Hi-C–based chromosome-level scaffolding

Hi-C raw reads were filtered using Trimmomatic (v0.39;Bolger et al. 2014) with parameters LEADING:3 TRAILING:3 SLIDINGWINDOW:4:15 MINLEN:50 to remove adapters and low-quality bases. Clean Hi-C reads were aligned to the contig assembly using BWA-MEM2 (v0.7.17; Jung and Han 2022) with parameters -SP5M. Invalid Hi-C read pairs (unmapped, multi-mapped, PCR duplicates) were removed using Juicer (v1.6;Durand et al. 2016), retaining valid pairs for downstream analyses. Based on the principle that cis-interactions (intra-chromosomal) are stronger than trans-interactions (inter-chromosomal), the 3D-DNA pipeline (v1.0; Dudchenko et al. 2017) was used to cluster, order, and orient contigs into scaffolds. Hi-C interaction heatmaps were visualized and manually curated with Juicebox (v2.13.07; Durand et al. 2016).

Genome quality assessment

BUSCO assessment was performed using BUSCO (v5.1;Seppey et al. 2019) based on the monocot database *liliopsida_odb10* (which contains 1,614 single-copy orthologous genes) to evaluate the completeness of the gene regions. Read mapping rates were calculated by aligning clean Illumina reads to the assembled genome using BWA (v0.7.17Li and Durbin 2009). Mapping statistics, including mapping rate, mean sequencing depth, and genome coverage (defined as the proportion of bases covered at ≥ 1× or ≥ 5× depth), were obtained using SAMtools (v1.10; Li et al. 2009). SNP and InDel validation was performed with the HaplotypeCaller module in GATK (v3.8; McKenna et al. 2010) to detect SNPs and InDels across the genome.The ratio of heterozygous to homozygous variants was then used to assess the accuracy of the assembly.

Genome annotation

Repetitive sequence annotation

Repetitive sequences were identified by combining both homology-based and de novo prediction approaches. For homology-based prediction, RepeatMasker (open-4.0.9; Tarailo-Graovac and Chen 2009) and RepeatProteinMask (open-4.0.9) were used together with the RepBase database (v26.03; Jurka et al. 2005) to annotate known transposable elements (TEs). For de novo prediction, RepeatModeler (open-1.0.11; Flynn et al. 2020) and LTR-FINDER (v1.0.5; Xu and Wang 2007) were employed to construct a species-specific repeat library, which was then used by RepeatMasker for TE annotation. Tandem Repeats Finder (TRF, v4.09; Benson 1999) with parameters of 2, 5, 7, 80, 10, 50 and 2000 was used to identify tandem repeats.

Protein-coding gene annotation

Three complementary strategies were integrated to predict gene structures.

De novo prediction: *Arabidopsis thaliana* gene models were used to train Augustus (v3.4.0; Stanke et al. 2006), GlimmerHMM (v3.0.4; Majoros et al. 2004), and Genscan (v1.0; Burge and Karlin 1997) for de novo gene prediction.

Homology-based prediction: Protein sequences from three closely related orchid species — *Platanthera zijinensis*, *Platanthera guangdongensis*, and *Dendrobium nobile* — were aligned to the *Ludisia discolor* genome using Exonerate (v2.4.0; Slater and Birney 2005) to identify homologous genes.

Transcriptome-assisted prediction: PacBio Iso-Seq full-length transcripts and Illumina RNA-seq short reads were aligned to the genome using GMAP (v2020-10-24; Wu and Watanabe 2005) and StringTie (v2.1.1;Pertea et al. 2015) to reconstruct transcripts. Open reading frames (ORFs) were predicted with TransDecoder (v5.5.0; <https://github.com/TransDecoder/TransDecoder>). The three sets of predictions were then integrated into a non-redundant gene set using MAKER (v3.00;Holt and Yandell 2011). For functional annotation, protein sequences were aligned to the SwissProt (v2024-05), TrEMBL (v2024-05), NR (NCBI non-redundant protein database), KEGG (v109), GO (v2024-05), and InterPro (v97.0) databases using BLASTP (v2.14.1;Altschul et al. 1990) with an E-value cutoff of 1e−5.

Non-coding RNA annotation

tRNAs were predicted based on secondary structure characteristics using tRNAscan-SE (v1.3.1;Lowe and Eddy 1997). rRNAs were identified by aligning the *Dendrobium officinale* rRNA sequences to the *Ludisia discolor*  genome using BLASTN (v2.14.1; E-value < 1e−10). miRNAs and snRNAs were annotated using the Rfam database (v14.1;Griffiths-Jones et al. 2005) and INFERNAL (v1.1.4;Nawrocki et al. 2009) through covariance model searches.

Comparative genomic analysis

Gene family clustering

Protein sequences from ten species—including *Ludisia discolor* and nine other Orchidaceae or monocot species (*Zea mays*, *Platanthera zijinensis*, *Platanthera guangdongensis*, *Dendrobium nobile*, *Vanilla planifolia*, *Apostasia shenzhenica*, *Dendrobium catenatum*, *Dendrobium chrysotoxum*, and *Dendrobium thyrsiflorum*)—were used for gene family clustering with OrthoFinder (v2.5.4;Emms and Kelly 2019). Single-copy orthologous gene families were identified by selecting families containing only one gene per species with amino acid length ≥ 100.

Phylogenetic tree construction and divergence time estimation

Single-copy orthologous genes were aligned using MUSCLE (v3.8.1551; Emms and Kelly 2019).The alignment results were concatenated into a supermatrix, and a maximum likelihood (ML) phylogenetic tree was constructed using RAxML (v8.2.12;Stamatakis 2014) with the GTR+GAMMA model and 1,000 bootstrap replicates. Divergence times were estimated with the mcmctree program in the PAML package (v4.9j; Yang 2007) and r8s (v1.8.1; Sanderson 2003). Calibration points were obtained from the TimeTree database (v5.0; Hedges et al. 2006): the divergence time between *Zea mays* and Orchidaceae was set at 119.2 Mya (95% CI: 110.2–124.4 Mya); the divergence time between *Vanilla planifolia* and other orchid species was set at 77.2 Mya (95% CI: 71.5–84.8 Mya).

Gene family expansion and contraction

CAFE (v5.0; De Bie et al. 2006) was used to analyze gene family expansion and contraction based on a birth–death model. The ML phylogenetic tree and gene family sizes were used as input.Significant expansions or contractions were identified at *P* < 0.05 (FDR-corrected Q < 0.05).GO and KEGG enrichment analyses of expanded and contracted families were performed using clusterProfiler (v4.6.0; Wu et al. 2021) with Q < 0.05.

Positive selection gene analysis

The branch-site model in PAML (v4.9j; Yang 2007) was used to detect positively selected genes (PSGs) in the *Ludisia discolor*  lineage. Two hypotheses were compared: H0 (no positive selection, ω ≤ 1) and H1 (positive selection, ω > 1). Likelihood ratio tests (LRT) were used to assess significance (*P* < 0.05), and genes with ω > 1 and *P* < 0.05 were identified as PSGs. GO and KEGG enrichment analyses of PSGs were conducted as described above.

Genome sequencing and survey analysis

Illumina short-read sequencing quality

To evaluate the genomic characteristics of *Ludisia discolor* prior to de novo assembly, paired-end (PE150) Illumina sequencing data were generated from 300–500 bp insert libraries. A total of 307,039,218 raw reads (46.06 Gb) were obtained. After quality filtering using SOAPnuke (v2.1.0), 299,037,606 clean reads (44.86 Gb) were retained. The sequencing quality of the clean reads was high: the Q20 values (base quality ≥ 20) of R1 and R2 were 99.3 % and 99.1 %, and the Q30 values (base quality ≥ 30) were 97.2 % and 96.4 %, respectively. The GC content of both read pairs remained stable at 35.5 %, and the absence of significant GC bias was an important factor ensuring the reliability of genome assembly (Huang et al. 2009). To evaluate potential exogenous contamination, 10,000 randomly selected clean read pairs were aligned to the NCBI NT database using BLASTN (v2.7.1; Altschul et al. 1990). The results showed that sequences from *Ludisia discolor* accounted for the highest proportion (4.3 %), followed by those matching closely related Orchidaceae species, including *Anoectochilus emeiensis* (1.08 %), *Goodyera schlechtendaliana* (0.77 %), *Dendrobium catenatum* (0.51 %), and *Pleione formosana* (0.42 %). No matches to bacteria, fungi, or other non-plant organisms were detected, confirming the absence of significant exogenous contamination and ensuring data purity for downstream analyses.

K-mer–based genome feature analysis

K-mer analysis (K = 17) was performed using GCE (v1.0.2;Liu et al. 2013) to estimate genome size, heterozygosity, and repeat content, which are key parameters for designing de novo assembly strategies. The genome size was estimated according to the Lander–Waterman algorithm (Lander and Waterman 1988):


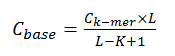

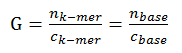


where *nBase* and *nK-mer* represent the total number of bases and K-mers, and *Cbase* and *CK-mer* are the expected base and K-mer coverage depths.。

The frequency distribution of K-mer coverage depth follows a Poisson distribution.


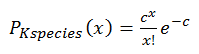


The probability function of the number of K-mer species for a genome with heterozygosity and repeats is:

${}$
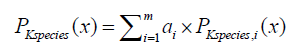


and the probability function of the number of K-mers is:

${}$
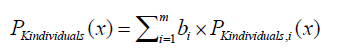


where $\boldsymbol{P}_{\text{Kspecies}}\boldsymbol{(x)}$and $\boldsymbol{P}_{\text{Kindividuals}}\boldsymbol{(x)}$correspond to Poisson and modified Poisson distributions with expected coverage depth $\boldsymbol{C}_{\boldsymbol{i}}$, respectively.

Assembly quality validation

Three complementary approaches were employed to assess the quality of the assembled genome.

BUSCO evaluation :Genome completeness was assessed using BUSCO (v5.1; Seppey et al. 2019) with the monocot dataset *liliopsida_odb10*, which includes 1,614 single-copy orthologous genes.

The results showed that 94.55% (1,526) of BUSCO genes were complete, of which 93.25% were single-copy and 1.30% were duplicated; 0.99% (16) were fragmented, and 4.46% (72) were missing. This level of completeness is comparable to other high-quality orchid genomes, such as *Phalaenopsis equestris* (93.8% complete BUSCO genes; Cai et al. 2015), indicating a high degree of assembly completeness in gene regions.

Read mapping and coverage: Clean Illumina reads were aligned to the chromosome-level assembly using BWA (v0.7.17; Li and Durbin 2009), yielding a mapping rate of 98.26%. The average sequencing depth was 28.9×, with 98.31% of the genome covered at ≥ 1× and 93.73% covered at ≥ 5×. These values indicate high assembly continuity with no significant gaps.

SNP and InDel detection: GATK (v3.8; McKenna et al. 2010) was used to detect 9,718,867 single-nucleotide polymorphisms (SNPs) and 1,077,114 insertions/deletions (InDels) across the genome.

Among these, 98.87% (9,609,350) of SNPs were heterozygous, consistent with the heterozygosity rate (0.63%) estimated from K-mer analysis, while homozygous SNPs accounted for only 1.13% (109,517). Similarly, 98.59% (1,061,913) of InDels were heterozygous, and 1.41% (15,201) were homozygous. The low proportion of homozygous variants confirms the high accuracy of the assembly and indicates the absence of systematic errors.

Genome annotation

Repetitive sequence annotation: A combination of homology-based and de novo prediction strategies was used to annotate repetitive elements, enabling detection of both known and species-specific transposable elements (TEs).

A total of 493.73 Mb of repetitive sequences were identified, accounting for 70.9% of the 696.37 Mb genome.

Tandem repeats: Tandem Repeats Finder (TRF v4.09; Benson 1999) identified 62.16 Mb of tandem repeat sequences, representing 8.93% of the genome. These repeats, including microsatellites and minisatellites, are often associated with genomic instability and phenotypic variation (Tautz and Schlötterer 1994).

Homology-based TE annotation: Using RepeatMasker (open-4.0.9; Nishimura 2000) and RepeatProteinMask with the RepBase database (v26.03;Jurka et al. 2005), 23.80 Mb (3.42%) and 74.49 Mb (10.7%) of TE sequences were annotated, respectively.

De novo TE annotation: De novo repeat library construction with RepeatModeler (open-1.0.11; Flynn et al. 2020) and LTR-FINDER (v1.0.5; Xu and Wang 2007) yielded 478.51 Mb of TE sequences, accounting for 68.71% of the genome. This represented the largest contribution to repeat detection, reflecting a high proportion of novel or lineage-specific TEs in *Ludisia discolor*.

TE classification: Long terminal repeat retrotransposons (LTR-RTs) were the most abundant class, spanning 358.25 Mb (51.44% of the genome), followed by DNA transposons (70.99 Mb, 10.19%), long interspersed nuclear elements (LINEs; 24.62 Mb, 3.54%), and short interspersed nuclear elements (SINEs; 1.21 Mb, 0.17%). The predominance of LTR-RTs is consistent with observations in other orchids, such as *Dendrobium officinale* (45.2% LTR-RTs; Yan et al. 2015), indicating that LTR-RT expansion has played a key role in shaping the *Ludisia discolor* genome.

Protein-coding gene annotation

Protein-coding gene prediction integrated three complementary approaches: de novo prediction, homology-based prediction, and transcriptome-assisted prediction. These predictions were then merged into a non-redundant gene set using MAKER (v3.00 https://hpc.nih.gov/apps/maker.html).

De novo prediction

Augustus (v3.4.0; Stanke et al. 2006), GlimmerHMM (v3.0.4; Majoros et al. 2004), and Genscan (v1.0)were trained with monocot gene models to predict 11,858, 37,257, and 2,930 genes, respectively.

Augustus and GlimmerHMM tended to identify shorter genes (average lengths 2,364 bp and 1,389 bp), whereas Genscan captured longer and structurally more complex genes (average length 14,088 bp), highlighting the benefit of combining multiple prediction tools.

Homology-based prediction

Exonerate (v2.4.0; Slater and Birney 2005) was used to align protein sequences from three closely related orchid species—*Platanthera zijinensis*, *Platanthera guangdongensis*, and *Dendrobium nobile*—to the *Ludisia discolor*  genome, predicting 27,847, 26,127, and 26,455 genes, respectively. These genes showed moderate lengths (average 6,215–8,255 bp) and exon numbers (3.48–3.88 exons per gene), reflecting evolutionary conservation of gene structure.

Transcriptome-assisted prediction

RNA-seq data from roots, stems, leaves, and flowers were aligned to the genome and assembled using GMAP (2020-10-24; Wu and Watanabe 2005) and StringTie (v2.1.1; Pertea et al. 2015), reconstructing 11,770 full-length transcripts. TransDecoder (v5.5.0; Tang et al. 2015) identified ORFs with the longest average gene length (15,529 bp) and highest exon count (6.27 exons per gene), reflecting the capture of alternative splicing isoforms.

Integrated gene set

A total of 20,552 non-redundant protein-coding genes were identified, with an average gene length of 11,000 bp, mean CDS length of 1,172 bp, and an average of 5.18 exons per gene.

These values are comparable to those reported in *Dendrobium officinale* (22,012 genes, 5.2 exons per gene; Yan et al. 2015), supporting the reliability of the annotation.

Functional annotation

By aligning the predicted protein sequences against seven public databases, functional annotations were obtained for 19,237 genes (93.6%). Annotation coverage was highest in the NR (NCBI non-redundant), TrEMBL, and KEGG databases, at 93.43%, 93.23%, and 92.41%, respectively. Additionally, 14,575 genes (70.92%) were annotated in SwissProt (Bairoch and Apweiler 2000) and 15,115 genes (73.55%) in InterPro (Zdobnov and Apweiler 2001), providing detailed information on protein structure and function.

Non-coding RNA annotation

Non-coding RNAs (ncRNAs) were annotated based on structural features and sequence conservation

miRNA:Using the Rfam database (v14.1; Griffiths-Jones et al. 2005) and INFERNAL (v1.1.4; Nawrocki et al. 2009), 2,754 miRNA genes were identified, with an average length of 269.6 bp and a total length of 742,526 bp, accounting for 0.11% of the genome. The abundance of miRNAs suggests complex regulatory networks controlling growth, development, and stress responses in *Ludisia discolor* (Bartel 2009).

tRNA: tRNAscan-SE (v1.3.1; Lowe and Eddy 1997) identified 1,300 tRNA genes with an average length of 76.0 bp, consistent with the conserved cloverleaf structure, and a total length of 98,751 bp (0.01% of the genome).

rRNA: BLASTN-based alignment to *D. officinale* rRNA sequences identified 2,032 rRNA genes, including 377 18S rRNA genes (average length 1,764 bp), 374 28S rRNA genes (3,981 bp), and 1,281 5S rRNA genes (115 bp). The total rRNA length was 2,300,965 bp (0.33% of the genome), reflecting a high copy number of rRNA operons required for ribosome biogenesis (Warner, 1999).

snRNA: Using Rfam and INFERNAL, 63 snRNA genes were annotated, including 22 CD-box snRNAs, 1 HACA-box snRNA, and 40 splicing-associated snRNAs. These snRNAs are core components of the spliceosome and mediate pre-mRNA splicing (Will and Lührmann 2011). No scaRNAs (small Cajal body–specific RNAs) were detected, suggesting potential lineage-specific loss or divergence of this ncRNA class in *Ludisia discolor*.

References:

Altschul SF, Gish W, Miller W, Myers EW, Lipman DJ (1990) Basic local alignment search tool. Journal of molecular biology 215 (3):403–410.doi:10.1016/s0022-2836(05)80360-2

Bairoch A, Apweiler R (2000) The SWISS-PROT protein sequence database and its supplement TrEMBL in 2000. Nucleic Acids Res 28 (1):45–48.doi:10.1093/nar/28.1.45

Bartel DP (2009) MicroRNAs: target recognition and regulatory functions. Cell 136 (2):215–233.doi:10.1016/j.cell.2009.01.002

Benson G (1999) Tandem repeats finder: a program to analyze DNA sequences. Nucleic Acids Res 27 (2):573–580.doi:10.1093/nar/27.2.573

Bolger AM, Lohse M, Usadel B (2014) Trimmomatic: a flexible trimmer for Illumina sequence data. Bioinformatics 30 (15):2114–2120.doi:10.1093/bioinformatics/btu170

Burge C, Karlin S (1997) Prediction of complete gene structures in human genomic DNA. Journal of molecular biology 268 (1):78–94.doi:10.1006/jmbi.1997.0951

Cai J, Liu X, Vanneste K, Proost S, Tsai WC, Liu KW *et al.* (2015) The genome sequence of the orchid Phalaenopsis equestris. Nat Genet 47 (1):65–72.doi:10.1038/ng.3149

Chen Y, Chen Y, Shi C, Huang Z, Zhang Y, Li S *et al.* (2018) SOAPnuke: a MapReduce acceleration-supported software for integrated quality control and preprocessing of high-throughput sequencing data. GigaScience 7 (1):1–6.doi:10.1093/gigascience/gix120

Cheng H, Concepcion GT, Feng X, Zhang H, Li H (2021) Haplotype-resolved de novo assembly using phased assembly graphs with hifiasm. Nature Methods 18 (2):170–175.doi:10.1038/s41592-020-01056-5

Chin CS, Peluso P, Sedlazeck FJ, Nattestad M, Concepcion GT, Clum A *et al.* (2016) Phased diploid genome assembly with single-molecule real-time sequencing. Nature methods 13 (12):1050–1054.doi:10.1038/nmeth.4035

De Bie T, Cristianini N, Demuth JP, Hahn MW (2006) CAFE: a computational tool for the study of gene family evolution. Bioinformatics 22 (10):1269–1271.doi:10.1093/bioinformatics/btl097

Dudchenko O, Batra SS, Omer AD, Nyquist SK, Hoeger M, Durand NC *et al.* (2017) De novo assembly of the Aedes aegypti genome using Hi-C yields chromosome-length scaffolds. Science (New York, NY) 356 (6333):92–95.doi:10.1126/science.aal3327

Durand NC, Robinson JT, Shamim MS, Machol I, Mesirov JP, Lander ES *et al.* (2016) Juicebox Provides a Visualization System for Hi-C Contact Maps with Unlimited Zoom. Cell Syst 3 (1):99–101.doi:10.1016/j.cels.2015.07.012

Emms DM, Kelly S (2019) OrthoFinder: phylogenetic orthology inference for comparative genomics. Genome biology 20 (1):238.doi:10.1186/s13059-019-1832-y

Flynn JM, Hubley R, Goubert C, Rosen J, Clark AG, Feschotte C *et al.* (2020) RepeatModeler2 for automated genomic discovery of transposable element families. Proc Natl Acad Sci U S A 117 (17):9451–9457.doi:10.1073/pnas.1921046117

Griffiths-Jones S, Moxon S, Marshall M, Khanna A, Eddy SR, Bateman A (2005) Rfam: annotating non-coding RNAs in complete genomes. Nucleic Acids Res 33 (Database issue):D121–124.doi:10.1093/nar/gki081

Hedges SB, Dudley J, Kumar S (2006) TimeTree: a public knowledge-base of divergence times among organisms. Bioinformatics 22 (23):2971–2972.doi:10.1093/bioinformatics/btl505

Holt C, Yandell M (2011) MAKER2: an annotation pipeline and genome-database management tool for second-generation genome projects. BMC Bioinformatics 12 (1):491.doi:10.1186/1471-2105-12-491

Huang S, Li R, Zhang Z, Li L, Gu X, Fan W *et al.* (2009) The genome of the cucumber, Cucumis sativus L. Nature genetics 41 (12):1275–1281.doi:10.1038/ng.475

Jung Y, Han D (2022) BWA-MEME: BWA-MEM emulated with a machine learning approach. Bioinformatics 38 (9):2404–2413.doi:10.1093/bioinformatics/btac137

Jurka J, Kapitonov VV, Pavlicek A, Klonowski P, Kohany O, Walichiewicz J (2005) Repbase Update, a database of eukaryotic repetitive elements. Cytogenet Genome Res 110 (1-4):462–467.doi:10.1159/000084979

Lander ES, Waterman MS (1988) Genomic mapping by fingerprinting random clones: a mathematical analysis. Genomics 2 (3):231–239.doi:10.1016/0888-7543(88)90007-9

Li H, Durbin R (2009) Fast and accurate short read alignment with Burrows-Wheeler transform. Bioinformatics 25 (14):1754–1760.doi:10.1093/bioinformatics/btp324

Li H, Handsaker B, Wysoker A, Fennell T, Ruan J, Homer N *et al.* (2009) The Sequence Alignment/Map format and SAMtools. Bioinformatics 25 (16):2078–2079.doi:10.1093/bioinformatics/btp352

Liu B, Shi Y, Yuan J, Galaxy Y, Zhang H, Li N *et al.* (2013) Estimation of genomic characteristics by analyzing k-mer frequency in de novo genome projects.doi:arxiv.org/abs/1308.2012

Lowe TM, Eddy SR (1997) tRNAscan-SE: a program for improved detection of transfer RNA genes in genomic sequence. Nucleic Acids Res 25 (5):955–964.doi:10.1093/nar/25.5.955

Majoros WH, Pertea M, Salzberg SL (2004) TigrScan and GlimmerHMM: two open source ab initio eukaryotic gene-finders. Bioinformatics 20 (16):2878–2879.doi:10.1093/bioinformatics/bth315

McKenna A, Hanna M, Banks E, Sivachenko A, Cibulskis K, Kernytsky A *et al.* (2010) The Genome Analysis Toolkit: a MapReduce framework for analyzing next-generation DNA sequencing data. Genome Res 20 (9):1297–1303.doi:10.1101/gr.107524.110

Nawrocki EP, Kolbe DL, Eddy SR (2009) Infernal 1.0: inference of RNA alignments. Bioinformatics 25 (10):1335–1337.doi:10.1093/bioinformatics/btp157

Nishimura D (2000) RepeatMasker. Biotech Software & Internet Report 1 (1-2):36–39.doi:10.1089/152791600319259

Pertea M, Pertea GM, Antonescu CM, Chang TC, Mendell JT, Salzberg SL (2015) StringTie enables improved reconstruction of a transcriptome from RNA-seq reads. Nat Biotechnol 33 (3):290–295.doi:10.1038/nbt.3122

Sanderson MJ (2003) r8s: inferring absolute rates of molecular evolution and divergence times in the absence of a molecular clock. Bioinformatics 19 (2):301–302.doi:10.1093/bioinformatics/19.2.301

Seppey M, Manni M, Zdobnov EM (2019) BUSCO: Assessing Genome Assembly and Annotation Completeness. Methods Mol Biol 1962:227–245.doi:10.1007/978-1-4939-9173-0_14

Slater GSC, Birney E (2005) Automated generation of heuristics for biological sequence comparison. BMC Bioinformatics 6 (1):31.doi:10.1186/1471-2105-6-31

Stamatakis A (2014) RAxML version 8: a tool for phylogenetic analysis and post-analysis of large phylogenies. Bioinformatics 30 (9):1312–1313.doi:10.1093/bioinformatics/btu033

Stanke M, Keller O, Gunduz I, Hayes A, Waack S, Morgenstern B (2006) AUGUSTUS: ab initio prediction of alternative transcripts. Nucleic Acids Res 34 (Web Server issue):W435–439.doi:10.1093/nar/gkl200

Tang S, Lomsadze A, Borodovsky M (2015) Identification of protein coding regions in RNA transcripts. Nucleic Acids Res 43 (12):e78.doi:10.1093/nar/gkv227

Tarailo-Graovac M, Chen N (2009) Using RepeatMasker to identify repetitive elements in genomic sequences. Current protocols in bioinformatics Chapter 4:4.10.11–14.10.14.doi:10.1002/0471250953.bi0410s25

Tautz D, Schlötterer (1994) Simple sequences. Current opinion in genetics & development 4 (6):832–837.doi:10.1016/0959-437x(94)90067-1

Walker BJ, Abeel T, Shea T, Priest M, Abouelliel A, Sakthikumar S *et al.* (2014) Pilon: an integrated tool for comprehensive microbial variant detection and genome assembly improvement. PloS one 9 (11):e112963.doi:10.1371/journal.pone.0112963

Will CL, Lührmann R (2011) Spliceosome structure and function. Cold Spring Harb Perspect Biol 3 (7).doi:10.1101/cshperspect.a003707

Wu T, Hu E, Xu S, Chen M, Guo P, Dai Z *et al.* (2021) clusterProfiler 4.0: A universal enrichment tool for interpreting omics data. Innovation (Cambridge (Mass)) 2 (3):100141.doi:10.1016/j.xinn.2021.100141

Wu TD, Watanabe CK (2005) GMAP: a genomic mapping and alignment program for mRNA and EST sequences. Bioinformatics 21 (9):1859–1875.doi:10.1093/bioinformatics/bti310

Xu Z, Wang H (2007) LTR_FINDER: an efficient tool for the prediction of full-length LTR retrotransposons. Nucleic Acids Res 35 (Web Server issue):W265–268.doi:10.1093/nar/gkm286

Yan L, Wang X, Liu H, Tian Y, Lian J, Yang R *et al.* (2015) The Genome of Dendrobium officinale Illuminates the Biology of the Important Traditional Chinese Orchid Herb. Mol Plant 8 (6):922–934.doi:10.1016/j.molp.2014.12.011

Yang Z (2007) PAML 4: phylogenetic analysis by maximum likelihood. Mol Biol Evol 24 (8):1586–1591.doi:10.1093/molbev/msm088

Zdobnov EM, Apweiler R (2001) InterProScan--an integration platform for the signature-recognition methods in InterPro. Bioinformatics 17 (9):847–848.doi:10.1093/bioinformatics/17.9.847
